# Single-particle cryo-EM: alternative schemes to improve dose efficiency

**DOI:** 10.1107/S1600577521007931

**Published:** 2021-08-26

**Authors:** Yue Zhang, Peng-Han Lu, Enzo Rotunno, Filippo Troiani, J. Paul van Schayck, Amir H. Tavabi, Rafal E. Dunin-Borkowski, Vincenzo Grillo, Peter J. Peters, Raimond B. G. Ravelli

**Affiliations:** a Maastricht Multimodal Molecular Imaging Institute, Division of Nanoscopy, Maastricht University, Universiteitssingel 50, Maastricht 6229 ER, The Netherlands; b Ernst Ruska-Centre for Microscopy and Spectroscopy with Electrons and Peter Grünberg Institute, Forschungszentrum Jülich, Jülich 52425, Germany; c CNR-Istituto Nanoscienze, Centro S3, Via G Campi 213/A, I-41125 Modena, Italy

**Keywords:** phase plate, multi-pass TEM, holography, ptychography, quantum sorter

## Abstract

Imaging of biomolecules by ionizing radiation, such as electrons, causes radiation damage which introduces structural and compositional changes of the specimen. The total number of high-energy electrons per surface area that can be used for imaging in cryogenic electron microscopy (cryo-EM) is severely restricted due to radiation damage, resulting in low signal-to-noise ratios (SNR). High resolution details are dampened by the transfer function of the microscope and detector, and are the first to be lost as radiation damage alters the individual molecules which are presumed to be identical during averaging. As a consequence, radiation damage puts a limit on the particle size and sample heterogeneity with which electron microscopy (EM) can deal. Since a transmission EM (TEM) image is formed from the scattering process of the electron by the specimen interaction potential, radiation damage is inevitable. However, we can aim to maximize the information transfer for a given dose and increase the SNR by finding alternatives to the conventional phase-contrast cryo-EM techniques. Here some alternative transmission electron microscopy techniques are reviewed, including phase plate, multi-pass transmission electron microscopy, off-axis holography, ptychography and a quantum sorter. Their prospects for providing more or complementary structural information within the limited lifetime of the sample are discussed.

## Abstract 2

Alternative single-particle cryo-EM schemes are reviewed, in order to improve dose efficiency for obtaining more or complementary structure information within the limited lifetime of the sample.

## 1. Introduction   

Cryogenic electron microscopy (cryo-EM) has become a powerful tool for structural biologists to study the structure–function relationships of their biomolecules of interest. Recent development of transmission electron microscopy (TEM) hardware has led to atomic resolution for single-particle cryo-EM (Nakane *et al.*, 2020 ▸; Yip *et al.*, 2020 ▸). For smaller (<100 kDa), more heterogeneous and non-symmetric samples, it is still a challenge to reach near-atomic resolution.

Although TEM has the power to image individual atoms, its performance is limited by the radiation sensitivity of aqueous biological specimens and therefore we need to minimize exposure to the electron beam. Radiation damage results in changes to the specimen due to knock-on damage and radiolysis (Henderson, 1995 ▸; Ravelli & McSweeney, 2000 ▸; Weik *et al.*, 2000 ▸; Williams & Carter, 2009 ▸; Karuppasamy *et al.*, 2011 ▸; Egerton, 2019 ▸). In TEM, the radiation dose is traditionally defined as charge density (C m^−2^) or electrons per unit area (e^−^ Å^−2^) (Williams & Carter, 2009 ▸). Here, we use the absorbed dose in gray (Gy), which is the radiation energy (J) absorbed per unit mass (kg) and relates to the beam parameters (electron energy, electron flux and shape), sample thickness and composition (Murray *et al.*, 2004 ▸; Egerton, 2021 ▸). Henderson proposed a dose limit of 20 MGy at which the diffracted intensity coming from a cryo-cooled crystal would be halved (Henderson, 1990 ▸), whereas Garman obtained an experimental value of 43 MGy (Owen *et al.*, 2006 ▸). Glaeser reported a fluence of 6 e^−^ Å^−2^ at 80 keV to cause significant damage to biological specimens (Glaeser, 1971 ▸), which can be calculated to correspond to a dose of 44 MGy using *Raddose-3D* (Bury *et al.*, 2018 ▸). Micro-electron diffraction is able to solve the macromolecules to atomic resolution with such a dose (Unwin & Henderson, 1975 ▸; Bücker *et al.*, 2020 ▸); however, many important biomolecules cannot be crystallized. Ultimately, one wishes to image each particle individually, as each particle itself will define one point within the multi-dimensional conformation space a macromolecule can adapt. This is done in single-particle analysis (SPA), with a fluence of, typically, 40 e^−^ Å^−2^ at 300 keV, which would correspond to 149 MGy. Dose-weighting schemes are nowadays routinely applied to account for the loss of high-resolution information at such high doses (Scheres, 2014 ▸; Grant & Grigorieff, 2015 ▸).

Because of the large amount of energy being deposited in the sample for a relatively limited fluence, cryo-EM imaging of biological samples results in low signal-to-noise ratio (SNR) images. A large number of images of identical molecules are required to boost the SNR. The SNR of a single image, *X*_sig_, is given by Henderson (1995 ▸) and Zhang *et al.* (2020 ▸),
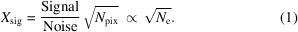
Here, *N*_pix_ is the number of pixels that corresponds to the area of one box containing the particle and *N*_e_ is the incident electron fluence. The SNR relates to the detectability of the whole protein, and is proportional to 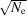. To increase the SNR, one could increase the signal, decrease the noise, or use a combination of both. The conventional way to increase the signal would be to increase the number of electrons *N*_e_. However, this will not really improve the SNR as the biological specimen will disintegrate upon exposure to the electron beam. For single-particle analysis, a large number of identical protein images is averaged to boost the SNR. The SNR is, therefore, also proportional to 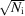, where *N*_i_ is the number of individual protein images.

Frozen-hydrated biological specimens consist predominantly of light elements that weakly modify the phase of the electron wave: typical SPA samples of biomolecules in solution are normally thin enough (∼20–100 nm) to be treated as weak phase objects. The phase shift of the exit wave is induced by the mean inner potential (MIP) of the sample which provides information about the electrostatic potential of the specimen. For weak phase objects, there is no amplitude contribution and the information transfer function becomes the contrast transfer function (CTF) multiplied by an aperture function and an envelope function (Reimer & Hohl, 2008 ▸; Williams & Carter, 2009 ▸),
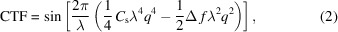
where *C*_s_ is the spherical aberration, λ is the wavelength, Δ*f* is the defocus and Δ*f* > 0 means underfocus, and *q* is the spatial frequency. At low defocus, there is very little transfer at low resolution, resulting in very poor contrast. The contrast can be improved by increasing the defocus, which will induce fast oscillations in the CTF. The information transfer is damped by the envelope function, *E*(*q*), which is the product of the envelope functions due to chromatic coherence [*E*_c_(*q*)] and the spatial coherence [*E*_s_(*q*)] of the electron source (Williams & Carter, 2009 ▸),
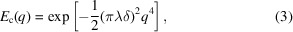


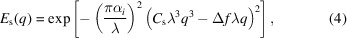
where δ is the defocus spread due to chromatic aberration. Therefore, increase of the defocus leads via *E*_s_(*q*) to more damping at high spatial frequency if the illumination semi-angle α_i_ is not small enough. Recently, Glaeser *et al.* (2021 ▸) pointed out that α can be as low as 1–2 µrad, and that therefore the damping of the CTF is not so much due to the spatial coherence of the electron source, so that defoci as high as 4 µm could be used. However, a high defocus results in information that becomes delocalized outside the field of view of the detector (Glaeser *et al.*, 2021 ▸). To this extent a small defocus value is still useful with the limited camera field that becomes further limited when very small pixel sizes are used for sub-2 Å structure reconstruction. Moreover, to reach atomic resolution reconstruction for biomolecules, aberration correction is essential. The axial coma aberration is the most limiting aberration in the atomic resolution regime, which can be corrected by software (Nakane *et al.*, 2020 ▸; Zivanov *et al.*, 2020 ▸) or by hardware such as by using a spherical aberration corrector (Yip *et al.*, 2020 ▸). Henderson (1995 ▸) represented the contrast of the image with a factor *C*, which varies from 0 to 1 (Henderson, 1995 ▸; Zhang *et al.*, 2020 ▸). This multiplicative factor *C* degrades the value of *X*_sig_ in equation (1), making it difficult to determine structures of smaller proteins (<100 kDa) to high resolution.

Here, we review alternative cryo-EM techniques for obtaining structural information of biomolecules in solution, mainly for weak phase objects, *e.g.* samples for SPA. SPA is carried out using conventional phase contrast TEM, whereas cryogenic scanning transmission electron microscopy (cryo-STEM) has been used for tomography of thicker biological samples (Wolf & Elbaum, 2019 ▸; Waugh *et al.*, 2020 ▸). Here, we discuss alternative techniques, exotic within the field of structural biology, which have different information transfer functions. We review TEM techniques, such as phase plate, multi-pass transmission electron microscopy (MPTEM) and off-axis holography, as well as STEM techniques, such as ptychography, and a quantum sorter. All these techniques will be discussed qualitatively in the next section. They provide alternative information transfer schemes, which, ultimately, could serve to improve the dose-efficiency in cryo-EM.

## 2. Alternative cryo-EM techniques to obtain structural information of biomolecules   

We review five alternative techniques in this section, namely a laser phase plate (Section 2.1), MPTEM (2.2), off-axis holography (2.3), ptychography (2.4) and a quantum sorter (2.5). Section 2.6 discusses the features, current status and perspectives of these techniques and compares these with conventional TEM.

### 2.1. Phase plate   

**Figure 1:**
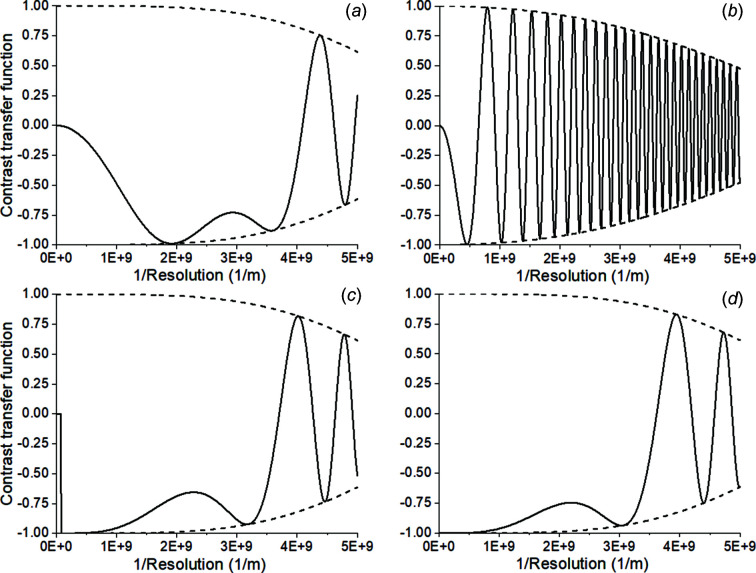
Contrast transfer function of (*a*) conventional TEM at Scherzer defocus, Δ*f*_Sch_ = 87 nm; (*b*) conventional TEM at defocus, Δ*f* = 1.2 µm; (*c*) with phase plate at defocus of 0.6Δ*f*_Sch_ = 52 nm with cut-on frequency at 1/25 nm^−1^; (*d*) holography at Gábor defocus, Δ*f*_Gabor_ = 49 nm. All have the illumination semi-angle set at 15 µrad.

One solution to improve information transfer over a wider range of spatial frequencies is to use a phase plate in the back focal plane of the objective lens. A phase plate can introduce a quarter-wave phase shift between transmitted and scattered electrons (Badde & Beimer, 1970 ▸; Danev *et al.*, 2009 ▸; Murata *et al.*, 2010 ▸; Danev *et al.*, 2014 ▸). Originating from light optics, Zernike phase plates were proposed and used to increase the phase contrast for weak phase objects by adding a π/2 phase shift to the diffracted beam (Zernike, 1942 ▸), altering the CTF into
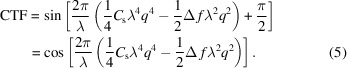
As the contrast of images is dominated by the low-frequency components, the form of the CTF is changed from sine to cosine which is crucial for the information transfer, in particular at low resolution. The cosine term allows one to record images close to focus, which reduces the damping of the envelope function [equation (4)] as shown in Fig. 1 ▸. The practical implementation of a phase plate for TEM, however, has been cumbersome. Most thin film phase plates and even electrostatic phase plates (Schultheiß *et al.*, 2006 ▸; Danev & Nagayama, 2008 ▸; Edgcombe *et al.*, 2012 ▸; Walter *et al.*, 2012 ▸; Danev *et al.*, 2014 ▸; Tavabi *et al.*, 2018 ▸; Verbeeck *et al.*, 2018 ▸; Schwartz *et al.*, 2019 ▸; Turnbaugh *et al.*, 2021 ▸) undergo electrostatic charging which results in poor reliability and a short working lifetime of the device, and distortion of the final image (Glaeser, 2013 ▸). The Volta Phase Plate (Danev *et al.*, 2014 ▸), which is commercially available, suffers from the fact that the phase shift is variable. Furthermore, it introduces inelastic scattering after the sample, resulting in precious electron loss. A phase plate which gives stable π/2 phase shift, and induces almost no electron loss and post-sample scattering, should be ideal for biological specimens.

Recently, an alternative approach was devised, based on electron–light interaction: the laser phase plate (Barwick & Batelaan, 2008 ▸). This laser-based phase plate still requires the ‘centering’ operation, *i.e.* shifting the unscattered beam to one of the antinodes of a laser standing wave. However, unlike for most other phase plates, such an alignment procedure should not cause detrimental charging, simply because there is no material along the beam path. This should make long-term operation more reliable. For the same reason, this device is free from inelastic scattering (Marko *et al.*, 2013 ▸) and information cut-off (Hettler *et al.*, 2016 ▸), hence allowing for ultimately efficient transfer for almost all spatial frequencies with only reduced contrast along the laser standing wave direction (Schwartz *et al.*, 2019 ▸).

The incoming charge particles will be affected by a ponderomotive force: a force the charged particles experience in a rapidly oscillating electromagnetic field. The ponderomotive force will induce a phase shift on the electron, as given by (Schwartz *et al.*, 2017 ▸)
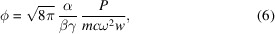
where α is the fine structure constant, *c* is the speed of light, *m* is the electron mass, β and γ are the electron’s relativistic factors, *P* is the beam power, ω is the laser angular frequency, and *w* is the size of beam waist. For 300 keV electrons, a π/2 phase shift requires a laser with an intensity of hundreds of GW cm^−2^ (Schwartz *et al.*, 2017 ▸). Meanwhile, in order to fit it into the column space of a TEM, the optical cavity needs to be small enough. Several different cavity designs were considered and a near-concentric Fabry-Pérot resonator was chosen to be applied within the TEM column (Müller *et al.*, 2010 ▸; Schwartz *et al.*, 2017 ▸).

**Figure 2:**
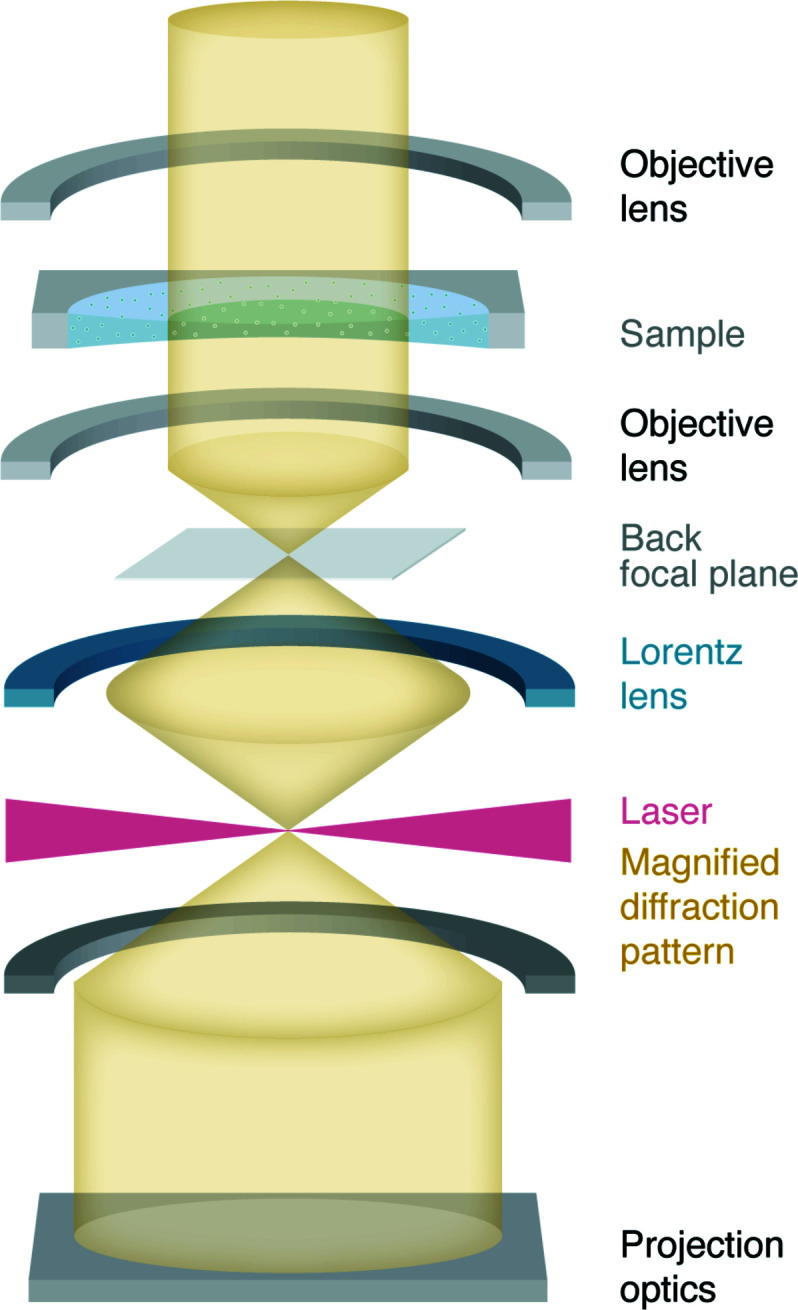
Schematic of a laser phase plate in a TEM. The high-power standing laser wave introduces a phase shift to the unscattered beam. A Lorentz lens after the objective lens is used for magnifying the back focal plane to reduce the constraint of the laser mode waist.

Lots of improvements have been achieved in obtaining a stable and high-power laser (Schwartz *et al.*, 2019 ▸; Turnbaugh *et al.*, 2021 ▸). First, a continuous-wave laser system has been designed and applied. Second, the laser intensity was increased from 43 GW cm^−2^ to above 120 GW cm^−2^ which gives a π/2 phase shift to 300 keV electrons with longer operating hours. Also, the laser mode waist has been decreased from 13 µm to 8.5 µm. A small mode waist is essential to obtain a smaller cut-on frequency (Danev & Nagayama, 2011 ▸), thereby maximizing the phase contrast at low spatial frequency. In addition, a transfer lens and a set of deflectors have been used to align the beam to the projection system. With this laser phase plate, a 3.8 Å 20S ribosome reconstruction could be obtained with just 4789 particles at a fluence of 50 e^−^ Å^−2^ at 300 keV (185 MGy) (Turnbaugh *et al.*, 2021 ▸). Figure 2 ▸ shows a schematic of the laser phase plate within a TEM column.

The mechanical and thermal stability of the laser system was one of the primary concerns, but recent SPA results obtained using the laser phase plate (Turnbaugh *et al.*, 2021 ▸) mitigated this doubt. The phase shift produced by this phase plate can in principle be adjusted by varying the laser intensity, which could be advantageous in situations such as the imaging of strong phase objects such as gold fiducial markers used for cryogenic electron tomography (Danev & Nagayama, 2011 ▸). However, changing the phase shift will most probably pose further technical challenges to the stability of the laser optics. An additional magnified diffraction plane must be created to match the laser spot size and to accommodate the space for the laser module. The addition of extra transfer lenses substantially increases the chromatic aberration, thereby limiting the spatial resolution that can ultimately be achieved (Turnbaugh *et al.*, 2021 ▸). This issue could possibly be resolved by redesigning the electron optics in future microscopes where the laser phase plate is implemented. All in all, this matter-free phase plate concept is both elegant and technically challenging, and involves serious add-ons to the microscope. We hope to hear much more about it in the years to come.

### 2.2. Multi-pass transmission electron microscopy   

Biological specimens embedded in a thin layer of vitreous ice are weak phase objects, which means that the phase shift of an electron passing through the sample is small. In order to increase contrast, one could increase the phase shift of the electrons. Let ϕ be the phase shift of the incoming electron beam after it passes through the specimen. If one could pass the same electron *m* times through the specimen, the accumulative phase shift would become *m*ϕ. This is exactly what is done in MPTEM.

**Figure 3:**
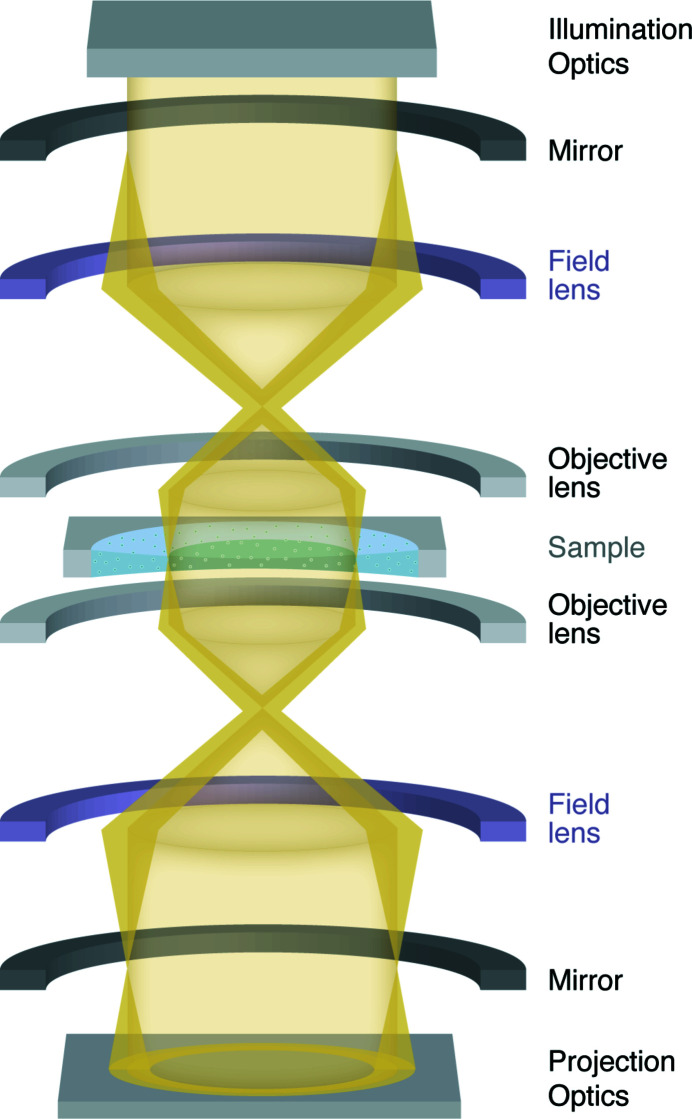
Schematic illustration of the MPTEM setup. The electron beam is generated by the illumination optics, passes through a gated mirror, goes through the sample, and bounces back and forth between the two gated mirrors (shown in black). After having passed through the sample *m* times, the beam is gated through the second mirror and enters the projection optics and is recorded by the detector.

MPTEM is being developed after the concept was first demonstrated in light microscopy (Juffmann *et al.*, 2016 ▸). The sample is placed in a self-imaging optical cavity. A pulse of light enters the self-imaging cavity through an in-coupling mirror and is scattered by the sample. Next, the formed image is reflected on a mirror and re-imaged onto the sample. After passing through the sample *m* times, the light leaves the cavity and is imaged using a microscope objective onto a camera. Analogous to light optical design, multi-pass microscopy was simulated (Juffmann *et al.*, 2017 ▸) and designed for TEM (Koppell *et al.*, 2019 ▸). Figure 3 ▸ shows a schematic view of a MPTEM setup (Koppell *et al.*, 2019 ▸).

In MPTEM, the signal is *m*-fold enhanced whereas the noise is unchanged. Thus, the SNR *X*_sig_ in equation (1) is *m*-fold enhanced as well, which is directly the main advantage of MPTEM (Juffmann *et al.*, 2017 ▸). For a given SNR, MPTEM will cause less radiation damage. For example, conventionally, we will have a SNR proportional to 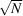 if we have *N* electrons as measurement probes. In MPTEM, we assume that there are *m* ^2^-fold less (*N*/*m* ^2^) incoming electrons passing through the specimen *m* times. As a result, the signal is proportional to *m*(*N*/*m* ^2^), which is actually lower than that for the conventional way. Fortunately, we care more about the SNR rather than the signal alone. As the noise level is proportional to the square root of the incoming electrons, it is 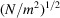. Thus, we have the same SNR as for conventional imaging,
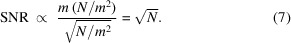
However, the damage to the sample is proportional to the product of electrons and the pass number 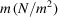 = 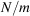, which is *m*-fold less than for a conventional TEM, while the SNR is constant.

The value of *m* is restricted by several factors. Firstly, once the phase shifts of the elastically scattered electrons have accumulated up to π, additional interaction will lead to a decrease of the phase contrast, thus lowering the signal. Simulations indicated that the SNR decreases at high interaction number, *m* (Juffmann *et al.*, 2017 ▸). Secondly, inelastic scattering restricts *m* as well. One needs to ensure that the total path length of the electron (*m* times sample thickness) in the specimen is smaller than the inelastic mean free path (IMFP) of the electron at the specific operating energy. A high interaction number induces high electron losses due to absorption and inelastic scattering, which reduces the efficiency of MPTEM. Simulation illustrates that when *m* ≃ 10, the MPTEM should have the optimal performance. In this case, the SNR will be three times higher at the same dose limit compared with conventional cryo-EM. This would enhance the resolution of a given sample (Koppell *et al.*, 2019 ▸), or allow for SPA to be performed on smaller biomolecules, or enable the processing of highly heterogenous samples (Henderson, 1995 ▸; Hall *et al.*, 2011 ▸; Zhang *et al.*, 2020 ▸; Su *et al.*, 2021 ▸).

However, various experimental challenges can be foreseen. Although the optical cavity is available for photons (Arnaud, 1969 ▸; Kolobov, 2007 ▸), a high-voltage electron mirror, which is a key component for MPTEM, does not yet exist. Meanwhile, the electron mirrors need to be well synchronized with the electron gun as well as with themselves in order to in-couple and out-couple the beam properly. A laser-triggered electron source fits the experiment best, since a continuous source would result in chromatic aberration (Koppell *et al.*, 2019 ▸). Moreover, the beam needs to interact with the same point of the specimen multiple times to have the correct phase accumulation. This requires very precise optics in the cavity. From all the techniques reviewed in this paper, MPTEM seems to be furthest away from practical applications.

### 2.3. Holography   

**Figure 4:**
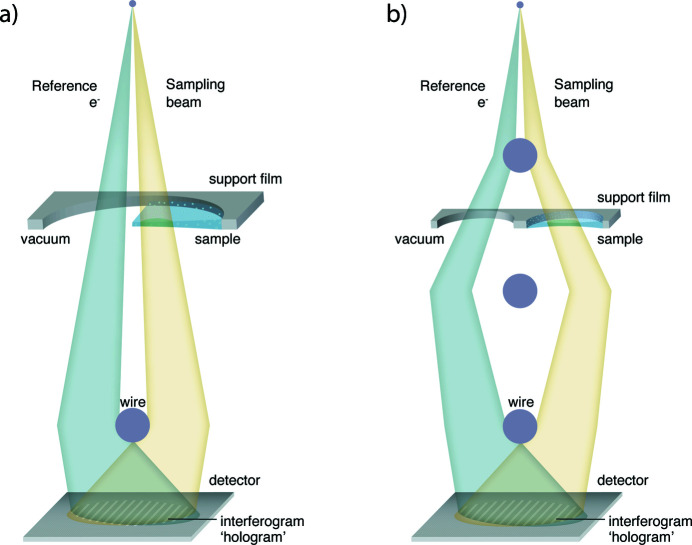
Schematic illustration of the off-axis electron holography setup. (*a*) Conventional off-axis electron holography. Half of the beam passes through the specimen (sampling beam) and the other half goes through the vacuum (reference beam). The two beams are next to each other, and the illuminated area can only be at the edge of the sample. Two beams are superimposed by the electron biprism, a positively charged wire, and interfere at the detector plane. (*b*) Split-illumination holography. The beam is split by condenser biprisms. The reference and sampling beam can go through two neighboring holes of the sample grid.

One of the main issues with conventional TEM is that only the amplitude of the exit wave is recorded, whereas the phase information is lost. We could reconstruct the whole exit wavefunction if we knew both the amplitude and phase. The phase shift of the exit wave provides information about the local variations of electrostatic potential in the sample. Full knowledge about the exit wavefunction could also benefit the study of biological objects, as it offers the possibility of correcting the residual aberration digitally by implementing virtually phase plates in software rather than in hardware (Winkler *et al.*, 2018 ▸). One way of obtaining the phase information is by interfering the exit wave with a reference plane wave. Dennis Gabor was awarded the Nobel Prize in Physics for his invention and development of the in-line holographic method (Gabor, 1949 ▸), in which the interfering reference and object waves shared the same optical axis. Möllenstedt and Düker proposed off-axis holography (Möllenstedt & Düker, 1956 ▸), in which the exit wave and reference wave are deflected and overlapped by an electron biprism [Fig. 4 ▸(*a*)]. The electron biprism consists of a positively charged wire. Here, we mainly focus on the off-axis holography technique.

In holography, by superimposing the exit wave with the reference plane wave, both the phase and amplitude of the exit wave can be resolved. The intensity of the interference pattern is given by (Simon *et al.*, 2003 ▸, 2008 ▸, Dunin-Borkowski *et al.*, 2019 ▸)
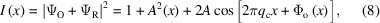
where Ψ_O_ is the exit-wave, Ψ_R_ is the reference plane wave, *A*(*x*) and Φ_o_(*x*) are the amplitude and phase of the exit wave, respectively, and *q*_c_ is the spatial frequency of the interference fringes. From equation (8), we know that the final intensity consists of a reference wave amplitude, an exit-wave amplitude and an exit-wave phase. To reconstruct the complex exit-wavefunction, one takes the Fourier transform of the hologram,
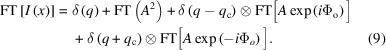
This function contains three bands: (i) the central band of the intensity distribution without phase information, δ(*q*) + FT(A^2^); (ii) the +1 sideband at *q* = *q*_c_; and (iii) the −1 sideband at *q* = −*q*_c_, which both contain the complete exit wave. Therefore, after the Fourier transform of the hologram, amplitude and phase distribution can be obtained from the inverse Fourier transform of either one of the two sidebands. The spatial resolution of the phase image is normally threefold better than the fringe spacing (Völkl & Lichte, 1990 ▸; Yamamoto *et al.*, 2010 ▸, 2021 ▸). It is essential to have fine interference fringes for high spatial resolution. However, this decreases the spatial coherence of the beam, and as a result the fringe contrast decreases.

Ru *et al.* (1991 ▸) proposed phase-shifting electron holography to overcome this problem. This approach takes a series of holograms with interference fringes shifted every image by a beam-tilt, allowing for the retrieval of both the amplitude and phase information of the objective wave from the periodically changing fringe intensity (Ru *et al.*, 1994 ▸). It is unnecessary to filter the signal in Fourier space, since phase-shifting electron holography is able to obtain high-resolution phase images without the loss of fringe contrast (Yamamoto *et al.*, 2021 ▸).

For biological applications, off-axis holography is able to enhance contrast from low spatial frequency to high spatial frequency as the phase contrast transfer function is in the form of a cosine (Simon *et al.*, 2008 ▸). This is in contrast to conventional TEM, where the contrast is enhanced by applying a high defocus at the cost of fast damping of CTF and loss of high spatial frequency signal. Off-axis holography has been applied to investigate ferritin (Kawasaki *et al.*, 1986 ▸; Harscher & Lichte, 1996 ▸; Weierstall & Lichte, 1996 ▸), flagella of bacteria (Aoyama & Ru, 1996 ▸), tobacco mosaic virus (TMV) (Aoyama & Ru, 1996 ▸; Weierstall & Lichte, 1996 ▸), T5 bacteriophage virus, the hexagonal-packed intermediate layer of bacteria, Semliki Forest virus, as well as collagen fibers and the surface layer of bacteria (Harscher, 1999 ▸; Lichte *et al.*, 2007 ▸; Simon *et al.*, 2008 ▸). Holography has also been used to study the electrical or magnetic field within a biological sample (Dunin-Borkowski *et al.*, 1998 ▸; Prozorov *et al.*, 2017 ▸).

There are several concerns regarding imaging biological specimens using holography. Firstly, the contrast of the images and the interference fringes can be poor, which has been attributed to the MIP and coherence of the electron beam, respectively. For cryo-EM, the biological specimen is located within a thin layer of vitreous ice and only induces a weak phase shift to the electron beam. To enhance the contrast, one could increase the sample thickness or use a higher dose, but these would induce more inelastic scattering and beam damage, respectively. The inelastically scattered electrons can reduce the contrast of the interference fringes as they are not coherent with the reference beam. High beam coherence requires a field emission gun (FEG) electron source, a small spot size, a small condenser aperture and a low gun extraction voltage (Dunin-Borkowski *et al.*, 2019 ▸).

Secondly, to obtain a reference beam, either vacuum or vitreous water without single particles is needed within the direct vicinity of the specimen for conventional electron holography. The required short distance between the sample and reference beams restricts the region of the sample that can be studied. In Fig. 4 ▸(*a*), we drew a hole of a typical quantifoil support which is often used for SPA. These holes of, for example, 1.2 µm width, are normally filled with biomolecules embedded within a thin layer of vitreous ice. For conventional holography to work, half of this hole should be empty to allow passage of the reference beam. The split-illumination electron holography [Fig. 4 ▸(*b*)] method involves inserting extra bi­prisms into the condenser system to allow for a much larger distance between sample and reference beam (Tanigaki *et al.*, 2012 ▸, 2014 ▸). In combination with the new sample preparation methods, such as the pinprinting of sample on specific parts of the grid (Ravelli *et al.*, 2020 ▸), split-illumination holography might become possible for a wider range of biological applications.

Due to the low contrast and resolution of the biological specimen obtained with holography images, only very few such experiments have been reported. For ice-embedded samples, there are several hurdles: the requirement of a nearby hole, the small differences between the MIP of a protein and its surrounding ice, and destruction of the contrast due to charging of the ice. Recently, low-energy (20 keV) in-line holography experiments have been reported on ice-embedded samples of TMV virions, T4 bacteriophages and erythrocruorin (Cheung *et al.*, 2020 ▸). They showed that the sample is clearly visible in thin ice (∼40 nm) and blurred in relatively thick ice (∼90 nm), and discuss the importance of reduced exposure times to prevent the washing out of interference fringes by mechanical or environmental factors. In addition, compared with the charge-coupled device (CCD) camera used in the previous experiments, direct electron directors have much better detective quantum efficiency (DQE) and modulation transfer function (MTF). This greatly improves the contrast of the interference fringes and reduces the phase error of the reconstructed electron wavefunction, and thus improves the dose efficiency (Chang *et al.*, 2016 ▸). Nowadays, with new sample preparation methods (Ravelli *et al.*, 2020 ▸), more stable microscopes, direct electron detectors, motion correction (Li *et al.*, 2013 ▸; Scheres, 2014 ▸) and additional approaches such as phase-shifting electron holography and split-illumination electron holography, off-axis electron holography might see a revival of interest that could complement SPA cryo-EM.

### 2.4. Ptychography   

Ptychography is a method where an image is computationally reconstructed from a series of diffraction patterns. It is based on the measurement of a matrix of correlated diffraction images as a function of probe position. It allows the reconstruction of both the phase and the amplitude of an object by reverse calculating it from the reciprocal space data. The basic principle of this technique was proposed more than 50 years ago (Hoppe, 1969 ▸) and it has been widely used in X-ray (Pfeiffer, 2018 ▸) and extreme ultraviolet (Seaberg *et al.*, 2014 ▸; Odstrcil *et al.*, 2016 ▸; Baksh *et al.*, 2020 ▸) studies in the past decades (Rodenburg & Maiden, 2019 ▸). Only recently, catalyzed by the introduction of fast direct electron counting pixelated detectors (MacLaren *et al.*, 2020 ▸, Schayck *et al.*, 2020 ▸), has cryogenic electron ptychography started to receive more attention.

**Figure 5:**
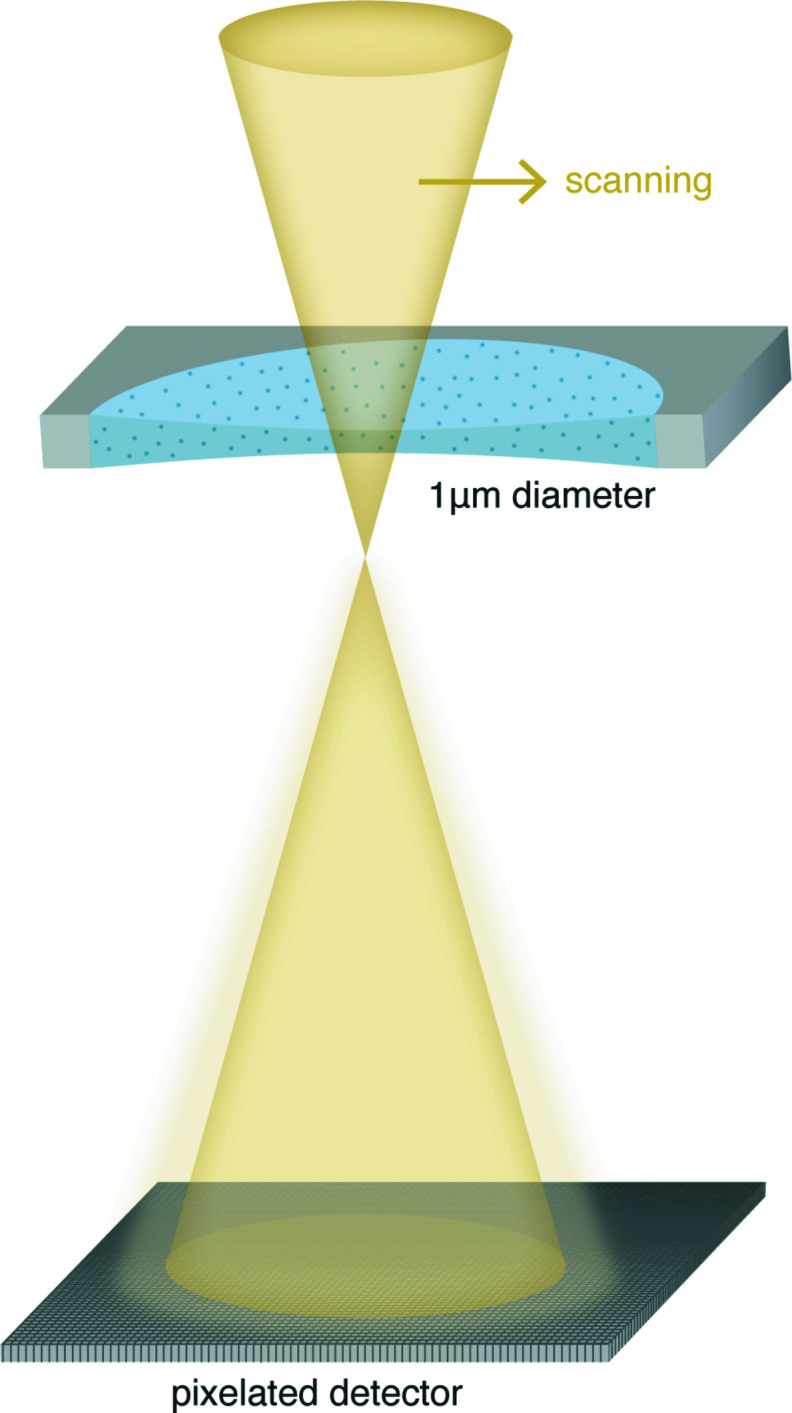
Schematic illustration of cryo-ptychography. A small convergent electron beam scans through the specimen (shown within 1 µm hole) at a certain defocus. A high-speed pixelated detector is used for recording diffractograms that are subsequently collected from partially overlapping sample positions.

In ptychography, a small scanning convergent electron beam is transmitted through the sample (Fig. 5 ▸). Ptychography has been stated to be the most dose-efficient approach compared with a few other STEM imaging modes (Yang *et al.*, 2015 ▸), including differential phase contrast (DPC). Since ptychography is able to directly reconstruct the phase of the specimen, it ought to be beneficial for the imaging of weak phase objects such as biological molecules. One important potential benefit of ptychography is that, depending on the spatial frequency range of interest, the optimal CTF can be chosen by varying the probe convergence semi-angle (Yang *et al.*, 2015 ▸; O’Leary *et al.*, 2021 ▸). This allows the retrieval of information at both low spatial frequency, such as morphology, and high frequency, such as macromolecular structures. One can design the experiment in such a way that the single-particle data collection is split into a few groups, each of which is taken with a significantly different probe convergence semi-angle to enhance the contrast for particular spatial frequencies. Since ptychography is an *in silico* imaging process, the reconstruction algorithm also plays a key role in the information transfer and, in fact, each algorithm possesses its own intrinsic contrast transfer function. For example, it was reported (O’Leary *et al.*, 2021 ▸) that the extended ptychographic iterative engine (ePIE) (Maiden & Rodenburg, 2009 ▸), the most commonly used iterative ptychography reconstruction algorithm, delivers a broader contrast transfer range compared with the single-side-band (SSB) method (Rodenburg *et al.*, 1993 ▸), a typical non-iterative algorithm for electron ptychography. Therefore, careful selection and optimization of the reconstruction algorithm seems vital to obtain high-contrast ptychographic phase images.

Like with any cryo-EM technique, the absolute total information transfer in ptychography will be limited by the total electron dose the sample can withstand. Recently, it has been experimentally demonstrated that electron ptychography can resolve sub-2 Å resolution on 2D materials using less than 400 e^−^ Å^−2^ (∼1.5 GGy) (Chen *et al.*, 2020 ▸). Also, last year, cryo-ptychographic reconstructed phase images were shown for virus particles that were inspected using a typical cryo-EM dose of ∼20 e^−^ Å^−2^ (∼75 MGy) down to nanometre resolution (Zhou *et al.*, 2020 ▸).

Simulations have shown that the use of a structured probe with random speckles can introduce strong fluctuations to the diffraction pattern which are particular helpful for ultra-low-fluence data as is the case in cryo-EM (Pelz *et al.*, 2017 ▸). It was predicted that ptychography would need two orders of magnitude fewer molecules imaged, compared with conventional defocus cryo-EM, to obtain the same target resolution within the same dose lifetime of the specimen (Pelz *et al.*, 2017 ▸). Similar probe shaping proposals have been carried out experimentally in STEM ptychography or its variant setups (Yang *et al.*, 2016 ▸; Broek *et al.*, 2019 ▸; Allars *et al.*, 2021 ▸) using thin-film-based phase masks. Alternatively, one can use electrostatic electron wave shaping devices (Verbeeck *et al.*, 2018 ▸; Tavabi *et al.*, 2020 ▸) to avoid profuse scattering from the thin-film masks.

There are a number of concerns regarding applying electron ptychography to biological imaging. The applicability of ptychography has been severely limited by sample thickness, and most of the previous experiments were performed on 2D materials. For samples that are too thick to be treated as a projected potential neglecting beam propagation (which is usually only a few nanometre), multislice ptychography approach has been proposed (Maiden & Rodenburg, 2009 ▸) and recently demonstrated for electrons (Chen *et al.*, 2021 ▸). This uses multiple slices to represent the sample and retrieve the structure of each slice separately. In practice, a monolayer of biomolecules within a well vitrified ice layer that is only slightly thicker than the macromolecules could work fine for normal ptychography.

Another concern is that the need for sufficient overlap between neighboring probe illumination areas will lead to different degrees of beam damage across the sample. The biological specimen inside the probe is going to be slightly damaged after the first diffraction pattern, and part of the illuminated area will be irradiated again for the second diffraction pattern. Even when a neighboring region is not directly illuminated by the probe itself, it might still suffer from beam damage due to electrostatic charging as well as damage spreading (Muller & Silcox, 1995 ▸; Riekel *et al.*, 2010 ▸) which will vary dynamically during the scanning of the beam. The gradual degradation and inconsistent quality of the sample may affect the result of the ptychographic reconstruction. Specific dose-weighting schemes, analogous to those developed for SPA, will have to be implemented for ptychography to correctly account for beam damage.

The signification reduction of the number of images to be averaged could improve throughput and resolution and could reduce the minimum particle size that could be studied. However, the current state-of-the-art pixelated detectors are still not fast enough for efficient cryo-ptychography experiments as they would take milliseconds to record one diffraction pattern. In order to increase the throughput further, a better detector with more pixels (fine sampling in reciprocal space would allow for coarser sampling in real space) and faster speed is keenly desired. An ultra-fast detector with 512 × 512 pixels and 100 kHz rate is currently under development (Ciston *et al.*, 2019 ▸). Ptychography datasets are four-dimensional and the reconstruction is usually computationally very expensive. The field of single-particle cryo-EM faced similar challenges less than a decade ago, and managed to deal with them in a spectacular way. The coming decade should reveal the true potential of cryo-ptychography for structural studies of biomolecules in solution.

### 2.5. Quantum sorter   

Cryo-EM images of thin biological samples contain high-resolution information about the structure of each imaged molecule. This, however, does not directly reveal the identity of each molecule. In SPA, the imaging is performed on purified macromolecular complexes: biochemical characterization should provide prior knowledge on what one can expect to see by cryo-EM. Often, impurities can be observed within the micrographs as well. Sometimes, these can be computationally sorted and identified *posteriori* by image processing (Su *et al.*, 2021 ▸). In cryogenic electron tomography, one looks at unique samples, such as lamella of infected dendritic cells (Berger *et al.*, 2021 ▸), in which molecules are identified visually (Nickell *et al.*, 2006 ▸) or computationally, and used for sub-tomogram averaging (STA). Both schemes rely on molecule identification *posterio*, after the tomographic data have been collected and analyzed. *A priori* localization of individual molecules might become possible one day by means of correlative light-electron microscopy (CLEM) (Morgan *et al.*, 1960 ▸; Hanein & Volkmann, 2011 ▸), once the resolution and sensitivity of cryogenic light microscopy becomes high enough as well as fully compatible with cryo-EM. Below, we will describe how electrons can be used to identify individual molecules *a priori*, by means of a quantum sorter.

The use of a quantum sorter is a novel idea that allows for dose-efficient protein discrimination in cryo-EM by the use of custom electron bases. Once electrons impinge on a given protein, the wavefunction of each electron contains all the information on the protein projection. Unfortunately, when their wavefunction collapses on the final detector, only a small part of that information is captured. Therefore, a large number of electrons must be accumulated in order to create an interpretable image of the electron wavefunction. The common concept of image is related to the spatial representation of the electron wavefunction. Mathematically speaking, every representation, namely every set of commuting observables, can be used to analyze a wavefunction, but some representations may be more convenient, depending on the problem one wants to address. While space representation is the one most commonly used, other representations could be more appropriate. One typical example is the angular basis, which includes the radial degree of freedom and orbital angular momentum (OAM) of the electron beam (Verbeeck *et al.*, 2010 ▸; McMorran *et al.*, 2011 ▸; Grillo *et al.*, 2017 ▸; Tavabi *et al.*, 2021 ▸), and allows one to improve the efficiency of the measurement by increasing the amount of information that can be accessed in the measurement.

**Figure 6:**
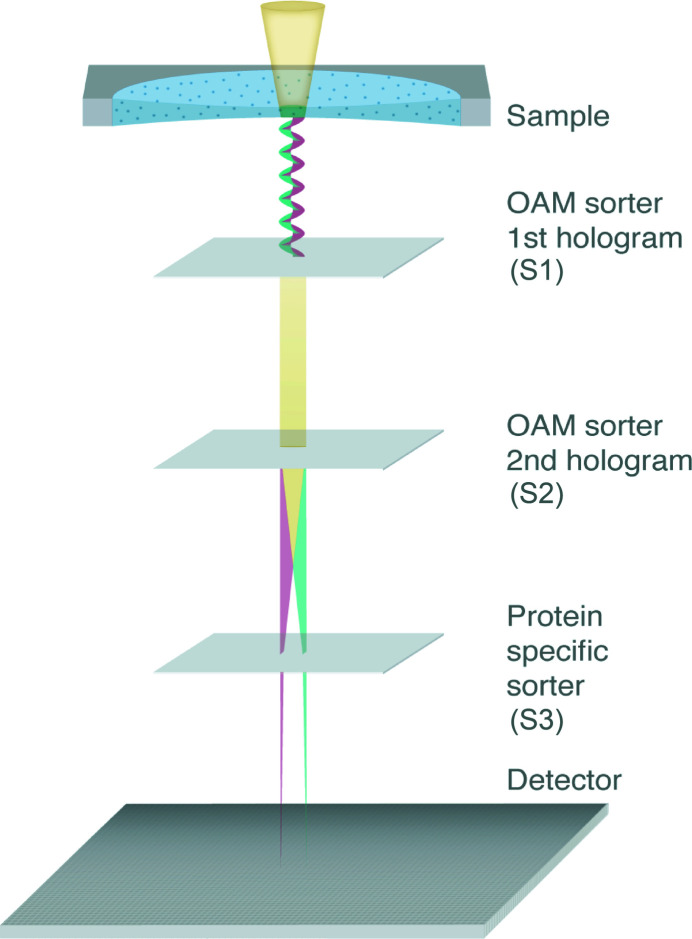
Scheme of the new electro-optical setup allowing optimal discrimination between proteins. An OAM sorter made by three elements performs the Cartesian-polar mapping. Electron vortex beams which contain information hidden in their OAMs are generated after passing through the sample in the specimen plane. The first two holograms (S1, S2) perform the OAM sorting, and the log-polar spectrum is further passed through another hologram (S3) and then through a cylindrical lens (not shown in the figure).

**Figure 7:**
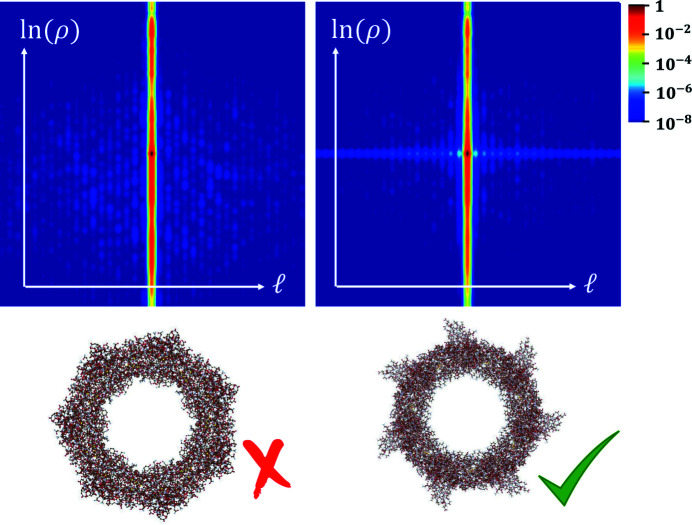
Comparison of the custom basis representation of proteins. The use of the optimized projection permits discrimination between the two models below. It can be appreciated that most of the information is contained in the *l* = 0 axis and in a restricted set of radial coordinates. Reproduced from Troiani *et al.* (2020 ▸) with permission from the American Physical Society.

Figure 6 ▸ represents the scheme of the electron microscope in the special configuration able to change the representation from a Cartesian to an angular basis. The device has already been demonstrated experimentally (Tavabi *et al.*, 2021 ▸). Two coupled phase holograms or two equivalent electrostatic phase elements are placed in the microscope. The sorter 1 (S1) transforms the OAM in exit wave from azimuthal coordinate to linear transverse coordinate, where the sorter 2 (S2) located in the diffraction plane of S1 corrects the phase distortion caused by S1 (Pozzi *et al.*, 2020 ▸). The only additional element described here is the projection element (S3) that permits to implement any measurement scheme. Figure 7 ▸ shows the new representation of the protein. Most of the information is located on the OAM axis (ℓ), thus producing a compact representation of the protein. For example, the presence or absence of rotational symmetry could be directly recognized on this axis. Given prior knowledge about the (low-resolution) structure of the model, the quantum sorter can be used to identify the presence of that molecule when a STEM beam is scanned over it. The capability to discriminate two different protein models using the quantum sorter was recently analyzed by Troiani *et al.* (2020 ▸) within the general framework of quantum state discrimination. For two given proteins, and for the two corresponding wavefunctions of the scattered electrons, one can derive the observable that maximizes the discrimination probability. Once the measurement scheme has been implemented, the approach consists of assigning each pixel of the image representation in the OAM space to the model A or B based on a maximum-likelihood criterion. The *a priori* probability for an electron to be measured in that pixel was calculated based on the two protein models. A merit factor was established by calculating how many electrons are required in order for the probability of identifying the protein to exceed a given threshold, say 95%. It was found by numerical simulation that an optimal measurement scheme could be constructed by modifying the angular basis by an appropriate projector on a new custom basis (Troiani *et al.*, 2020 ▸). When this strategy is applied, the number of electrons necessary for discrimination would be far less than 1 e^−^ Å^−2^ (3.7 MGy). Scanning the sample in advance with such a low fluence would allow identification of the location of specific proteins of interest, while preserving most of the sample lifetime for subsequent imaging by conventional means.

### 2.6. An overview   

We compared the illumination mode, desirable beam energy, state-of-the-art spatial resolution, the required fluence, and tolerance to thicker sample among the techniques listed above (Table 1 ▸). Features, current status and perspectives of these techniques are also included in Table 1 ▸. The listed illumination mode refers to the one most often used for that particular technique. For most techniques, other illumination modes are possible as well (Wolf *et al.*, 2014 ▸; Yasin *et al.*, 2018 ▸; Wolf & Elbaum, 2019 ▸; Allars *et al.*, 2021 ▸). While conventional cryo-EM has achieved atomic resolution for ferritin with a fluence 40 e^−^ Å^−2^ at 300 keV (148 MGy) (Nakane *et al.*, 2020 ▸; Yip *et al.*, 2020 ▸), smaller macromolecules and heterogeneous samples are still difficult to image. Some of the alternative techniques listed in Table 1 ▸ promise prospects to overcome these barriers.

## 3. Conclusion and outlook   

In this work, inspired by the huge interests in SPA from structural biologists, we outlined some alternative TEM and STEM imaging techniques and measurement methods, and discussed their dose efficiency. The radiation damage inflicted by high-energy electrons to the biological specimen is the major factor that limits the image SNR and the resolution. The so-called low-dose technique used in current conventional cryo-EM SPA uses the adjacent area of the sample to focus. This minimizes the beam damage to the area of interest before data acquisition, and has achieved good results. However, all SPA routines accept and computationally account for the oscillating character of the CTF, which dampens and even destroys the transfer of information at certain frequencies. Altering the information transfer function could improve the dose-efficiency at which we study biological specimens.

Phase plates make use of the electron-phase information that provides a more dose efficient approach for radiation-sensitive specimen. By using a phase plate in the back focal plane inside the TEM column, one can enhance the phase contrast of the image by altering the phase difference between the scattered and unscattered beam. Although a perfect Zernike-like phase distribution gives the optimal phase contrast, none of the current phase plates is able to give a delta function-like phase shift to the direct beam while unaffecting the scattered beam. A laser phase plate seems to be an ideal option as it offers a stable phase shift and it does not result in electron loss. With a 3.8 Å 20S ribosome reconstruction obtained with a 300 keV cryo-EM at a dose of 50 e^−^ Å^−2^ (185 MGy) (Turnbaugh *et al.*, 2021 ▸), we believe that the implementation of the laser phase plate for more cryo-EM SPA can be foreseen in the next few years.

The idea of MPTEM is to apply the transformation to the same electron multiple times to increase the SNR at a certain level of radiation damage. Another operating strategy of MPTEM is to reduce the radiation damage to the specimen while keeping SNR the same as in conventional cryo-EM. This can also be useful, since for SPA the frames above a certain dose have much less high-resolution information due to beam damage. However, a high-voltage electron mirror, the key component for MPTEM, does not yet exist. A low-energy (10 keV) proof-of-concept MPTEM is currently under construction (Koppell *et al.*, 2019 ▸), which could be the very first step to the application of MPTEM.

Holography and ptychography have compelling advantages in dose-efficient phase retrieval of the exit wave. Instead of imaging the specimen directly, holography records the interference pattern of the sample beam and reference beam. We are not aware of any recent reports of off-axis holography experiments on ice-embedded biological specimens, possibly due to the low contrast. A recent report of a low-energy in-line holography experiment pointed out that the ice thickness plays a key role in the contrast enhancement of the hologram (Cheung *et al.*, 2020 ▸). Together with additional approaches, *e.g.* phase-shifting holography and split-illumination holography, it could revitalize the application of holography on ice-embedded biological specimen. Ptychography reconstructs the exit-wave phase information computationally by recording a series of diffraction patterns. Cryo-ptychography has attracted researchers’ interest in recent years, and phase images of ice-embedded rotavirus have been reconstructed experimentally with the fluence of 20 e^−^ Å^−2^ (75 MGy) (Zhou *et al.*, 2020 ▸). With better illumination schemes and faster pixelated detectors actively being developed, cryo-ptychography for biological applications is likely to produce many more results in the years to come.

The use of a different basis compared with the traditional spatial representation of the electron wavefunction could open up entirely new concepts. We discussed the use of an angular basis, namely the orbital angular momentum, to identify the presence and location of known proteins within the sample, which here could be done in a highly dose-efficient manner. This might not only have potential to be applied to biomolecules in solution but also in the crowded cellular environment, such as for cryo-tomography of lamella of (infected) eukaryotic cells.

Conventional cryo-EM has seen tremendous developments over the last eight years, and the limits of SPA are still being pushed. Here we discussed a number of non-standard techniques, at different pioneering stages. In addition to the techniques discussed here, other techniques, such as aloof beam electron energy-loss spectroscopy (EELS) (Krivanek *et al.*, 2014 ▸; Egerton, 2015 ▸; Rez *et al.*, 2016 ▸), structured illumination with compressed sensing (Padgett & Boyd, 2017 ▸; Li *et al.*, 2018 ▸; Leary & Midgley, 2019 ▸), and adaptive optics with pixelated phase plates (Verbeeck *et al.*, 2018 ▸), could also have potential applications in imaging or characterization of biological specimens. TEM at low voltage reduces the radiation damage to the sample (Kaiser *et al.*, 2011 ▸; Egerton, 2019 ▸), and ultra-low-energy electron microscopy (LEEM) and eV-TEM at 0–30 eV have much less plasmonic and excitonic interactions, which results in almost no energy being deposited in the specimen (Geelen *et al.*, 2015 ▸; Neu *et al.*, 2021 ▸). TEM at such low acceleration voltage can, however, only achieve spatial resolution at a few nanometre range which will not allow for *de novo* structure determination. All in all, there are several alternative cryo-TEM and cryo-STEM schemes for obtaining more information during the limited lifetime of a biomolecule within the electron beam, thereby further pushing the limits of size, structural heterogeneity and resolution at which one can study the building blocks of life.

## Figures and Tables

**Table 1:** Comparison between different cryo-EM techniques

	Conventional cryo-EM	Laser phase plate	MPTEM	Holography	Ptychography	Q-sort
Illumination mode	TEM	TEM	TEM	TEM	STEM	STEM
Currently employed beam energies	100 keV to 300 keV	100 keV to 300 keV	10 keV	20 keV to 1.2 MeV	30 keV to 300 keV	100 keV to 300 keV
State-of-the-art spatial resolution and required fluence	3D reconstructions at atomic resolution (1.2 Å)	3D reconstructions at near-atomic resolution (3.8 Å)	PSF is 4–7 nm at 10 keV according to the simulation	2D atomic resolution for structural imaging	2D deep sub-Å resolution (0.39 Å) with high dose, around 400 e^−^ Å^−2^ with a single image and no averaging at 2D atomic resolution	Nanometre localization accuracy << 1 e^−^ Å^−2^
	∼40 e^−^ Å^−2^ averaged by a few hundred thousand particles	∼50 e^−^ Å^−2^ averaged by a few thousand particles	∼10 to 100 e^−^ Å^−2^ based on electron fluence and number of passes	2D and 3D nanometre resolution for EM fields		
				Not experimentally optimized for low dose cases	2D nanometre resolution with cryo-EM dose, ∼20 e^−^ Å^−2^ for virus particle embedded in ice	
Tolerance to thicker sample†	+	+	−−	−	−	+
Features	Defocus used to enhance contrast	Laser induced electron phase shift	Contrast enhancement by phase shift accumulation	Direct phase contrast imaging without using a phase plate	Direct phase contrast imaging without using a phase plate	Electron wavefunction is represented in OAM
		Matter free phase plate	Lower absorbed dose compared to conventional cryo-EM at the same SNR	Exit-wavefunction retrieval	Image is computationally reconstructed	Aiming for specific measurement, *e.g.* protein identification
		Adjustable phase shift		Allows for a wide band of spatial frequency to be transferred while lower spatial frequency is better preserved	Phase information retrieval	Extra elements are needed in TEM column
		Extra Lorentz lens			Adjustable contrast transfer band depending on the optical setup and reconstruction algorithm	
		High-power laser unit			Both sample transmission function and the illumination probe are retrieved which allows for post correction of residual optical aberration	
Current status	Atomic resolution achieved with ferritin	High power continuous-wave laser available for 300 keV electrons	Only simulation and design for 10 keV MPTEM are available	Low dose cryo-holography has hardly been used so far for biological samples	Advanced reconstruction algorithms are being developed to account for multiple scattering	Protein discrimination can be done, according to simulation, with << 1 e^−^ Å^−2^
	2 Å resolution is becoming more routine	3.8 Å 20S ribosome ihas been achieved	No experimental data yet	Direct electron detectors start to be used for improving the fringe visibility and therefore the signal to noise ratio	Extremely high spatial resolution beyond the diffraction limit and extremely high precision only limited by thermal noise has been demonstrated for non-biological samples	
	Proteins < 50 kDa are still difficult	Laser system stability needs to be ensure			First attempt of ptychographic reconstruction of vitrified biological specimen has been performed	
		Redesign electron optics optimized for laser phase plate				
Perspectives	Smaller macro­molecules	Atomic resolution should be achievable	Reliable and stable electron mirrors are needed, also at high energy	Imaging setups could be designed which carefully optimize spatial resolution, field of view, and fringe contrast	It could become possible to obtain 3D near-atomic resolution reconstruction with much fewer particles compared to what is used by conventional TEM	Localization of points of interest within a sample
	Better sample preparation	Smaller proteins should become possible	Low energy MPTEM has no obvious benefit compares to current cryo-EM	Split-illumination holography	Specific dose-weighting schemes to account for beam damage	Use of prior information to guide data collection and processing
	Better detectors	Heterogeneous samples should become easier		Phase-shifting holography	Automated cryo-ptychography SPA data collection	Structured illumination of the sample provide entirely new data collection schemes
	Fewer particles rejected			Smart sample preparation optimized for holographic experiments	Fast pixelated detector (∼100 kHz)	
	Better treatment of heterogeneous samples			Automated cryo-holographic SPA data collection	Fast data processing	

† Tolerance to thicker sample: + higher tolerance for thicker sample; − requires very thin sample.
